# Immunohistochemistry for PTEN testing in HR +/HER2˗ metastatic breast cancer

**DOI:** 10.1007/s00428-025-04249-5

**Published:** 2025-09-11

**Authors:** Nicola Fusco, Elena Guerini-Rocco, Isabella Castellano, Umberto Malapelle

**Affiliations:** 1 Division of Pathology, European Institute of Oncology IRCCS, Milan, Italy; 2 Department of Oncology and Hemato-Oncology, University of Milan, Milan, Italy; 3 Pathology Unit, Department of Medical Sciences, City of Health and Science University Hospital, University of Turin, Turin, Italy; 4 Department of Public Health, University Federico II of Naples, Naples, Italy

**Keywords:** PTEN, Immunohistochemistry (IHC), Metastatic breast cancer (MBC), PI3K/AKT pathway, Next-generation sequencing (NGS)

## Abstract

The *PTEN* tumor suppressor regulates the *PIK3CA/AKT1* pathway, and its inactivation significantly contributes to tumorigenesis and progression in hormone receptor-positive/HER2-negative (HR + /HER2 −) metastatic breast cancer (MBC). In ~ 5% of these patients, PTEN loss, primarily due to gene deletions, leads to aberrant PI3K signaling and enhanced oncogenic potential. Findings from the CAPItello-291 study further establish *PTEN* together with *PIK3CA* and *AKT1* as a predictive biomarker for Capivasertib, a pan-AKT inhibitor, in these patients. Despite next-generation sequencing (NGS) being the most precise method for detecting gene losses, immunohistochemistry (IHC) offers some advantages, including accessibility, cost-effectiveness, and applicability when archival tissue is inadequate for NGS or when pre-analytical failure occurs. Notably, recent evidence supports a pragmatic IHC positivity criterion, defining PTEN deficiency as staining in less than 10% of tumor cells, regardless of intensity. In this manuscript, we provide a comprehensive overview of the clinical scenarios associated with PTEN IHC testing in HR + /HER2 − MBC, outline best practices to minimize the impact of pre-analytical and analytical variability, and propose a structured pathology report to standardize PTEN IHC evaluation in this context.

## Introduction

The *PTEN* tumor suppressor gene plays a Major role in regulating the phosphatidylinositol 3-kinase (PI3K) pathway and its oncogenic signaling [1]. In breast cancer, *PTEN* inactivation, mainly through gene deletions, occurs in 5% of hormone receptor (HR) +/HER2- metastatic breast cancer (MBC) and leads to tumorigenesis and tumor progression [2]. At present, three therapeutic options targeting the PI3K pathway are approved by both the Food and Drug Administration (FDA) and the European Medicines Agency (EMA) for the treatment of these patients [3]. Among these are Alpelisib and Inavolisib, which specifically inhibit the alpha isoform of PIK3CA, as well as Capivasertib, a first-in-class pan-AKT inhibitor [4–6]. Notably, Capivasertib has three potential biomarkers for patients’ selection: *PIK3CA*/*AKT1* activating mutations and *PTEN* loss of function [7]. In the CAPItello-291 study (NCT04305496), all these alterations were determined centrally by means of next-generation sequencing (NGS) with the use of the FoundationOne CDx assay on formalin-fixed paraffin-embedded (FFPE) tumor samples [6, 8].

In most pathology laboratories worldwide, immunohistochemistry (IHC) is widely used not only in diagnostic and predictive pathology but also as a surrogate for molecular classifications, due to its accessibility and cost-effectiveness [9–12]. For example, assessing PTEN protein loss through ancillary IHC analyses is routinely performed for gynecologic tumors [13, 14]. In an exploratory analysis presented at the San Antonio Breast Cancer Symposium 2024, the CAPItello-291 investigators used the Ventana PTEN SP218 IHC assay for PTEN testing [15]. Among samples with both NGS and IHC data, all cases with homozygous deletions or large PTEN rearrangements detected by NGS were classified as PTEN deficient by IHC (defined as < 10% staining in tumor cells); however, the reverse relationship was not consistently evident. Of note, PTEN-deficient tumors by IHC showed a promising progression-free survival (PFS) benefit with capivasertib plus fulvestrant compared to placebo plus fulvestrant (median PFS of 9.3 months versus 3.7 months; hazard ratio: 0.52.

While NGS should be considered the preferred testing method for breast cancer molecular testing [16, 17], IHC can still serve as a complementary approach, particularly in settings where NGS is not available, cost-prohibitive, or impractical due to limited tissue availability. Additionally, IHC offers a rapid and widely accessible method for assessing protein expression, which may provide clinically relevant insights, especially when timely treatment decisions are required. However, standardized protocols are essential to ensure accurate, reproducible, and clinically meaningful PTEN deficiency analysis by IHC in HR +/HER2 − MBC, minimizing variability and enhancing diagnostic accuracy.

## Recognizing the current challenges

PTEN is encoded by a single gene but multiple protein isoforms arise through alternative mRNA splicing or variations in translation initiation [18]. The most prevalent one, consisting of 403 amino acids (PTEN 1–403), is the primary form referred to in the literature on human cancers [19–21]. Numerous anti-PTEN monoclonal antibodies are available and have been variably used in breast cancer studies using IHC [22–42]. In addition to the variability in commercial PTEN assays, the broad spectrum of PTEN IHC staining patterns observed in breast cancer may pose diagnostic challenges [43]. Another potential issue is represented by the handling and processing of tissue samples to obtain reliable and reproducible IHC results [44, 45]. Pre-analytical variables—such as cold ischemic time, fixation duration, type of fixative used, and storage conditions—can dramatically affect PTEN antigen preservation [46–49]. Moreover, technical aspects of the staining procedure itself—including antigen retrieval methods, the selection of antibody clones, incubation times, temperature, and the detection system—play key roles in determining the intensity and quality of the staining [49–51]. In the post-analytical phase, interobserver variability might further complicate the picture [52–54]. These challenges underscore the need for rigorous quality control measures and the establishment of well-defined, standardized guidelines for PTEN IHC assessment specifically tailored to breast cancer [20, 55]. The implementation of strict standard operating procedures (SOPs) is pivotal in ensuring consistency and accuracy at every stage—from specimen collection and processing to staining and reporting [56–61]. Participation in external quality assessment (EQA) programs, such as JCI, UK NEQAS, and NordiQC, is highly recommended [62–67]. These programs provide benchmarking against standardized controls and help identify inconsistencies in staining and interpretation.

## Insights for assessing PTEN deficiency by IHC

### Scoring criteria

**Fig. 1:**
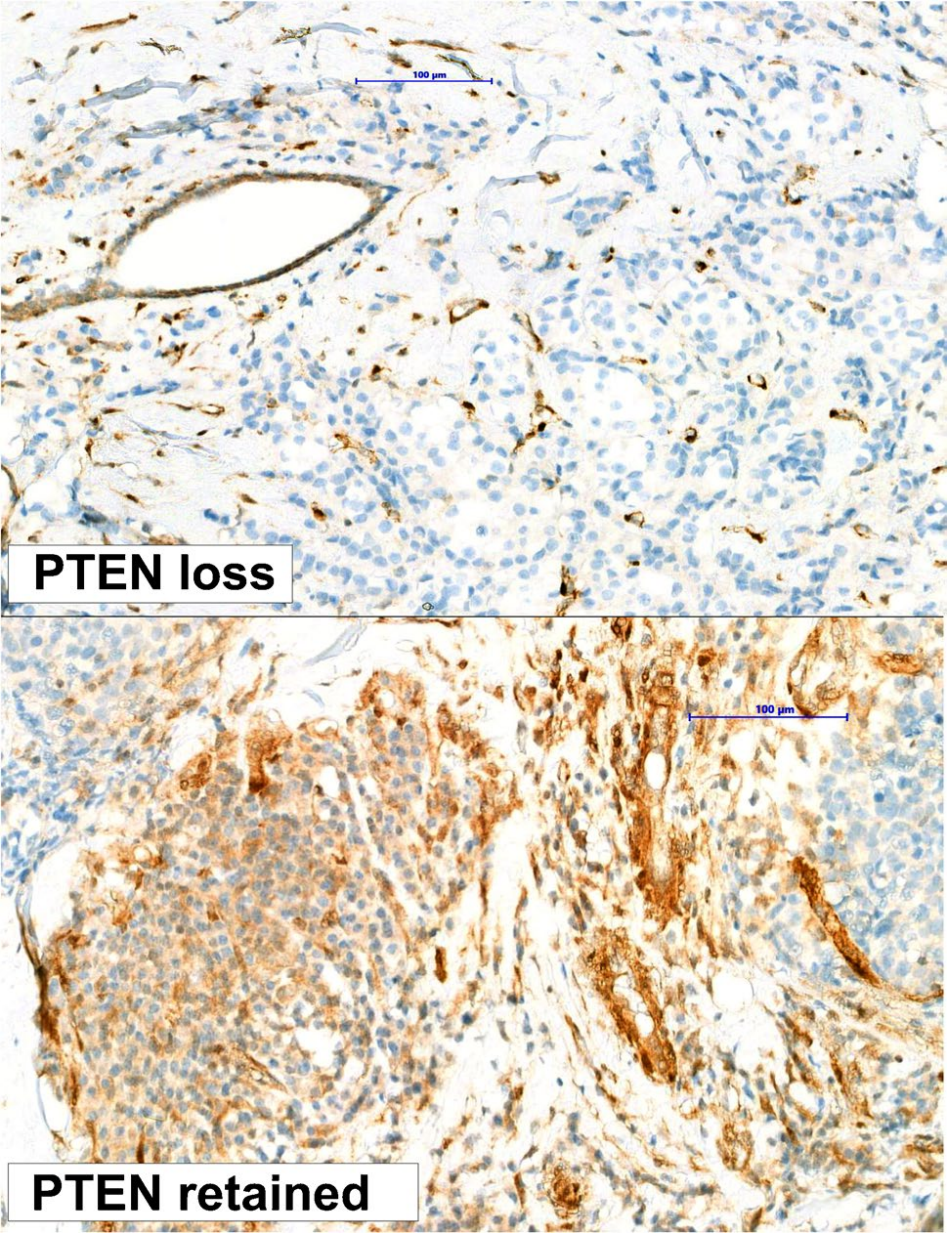
Representative micrographs of PTEN loss and PTEN retained patterns of expression in breast cancer using a 10% tumor cell positivity cutoff of any staining in the presence of acceptable internal controls

Accurate analysis of PTEN expression by IHC should focus on tumor cells, while breast glands should be used as internal positive controls (Fig. 1) [68]. The historically used scoring system in breast cancer employs a three-tier classification based on the ratio between normal and neoplastic cells [69]. However, this method does not account for tumor heterogeneity and lacks reproducibility. Based on insights from the CAPItello-291 investigators, a 10% tumor cell positivity cutoff of any staining in the presence of acceptable internal controls could be proposed to distinguish negative from positive cases (Fig. 1) [43, 70]. Further analytical validation using real-world data is required.

### Checklist and reporting of the results

**Fig. 2:**
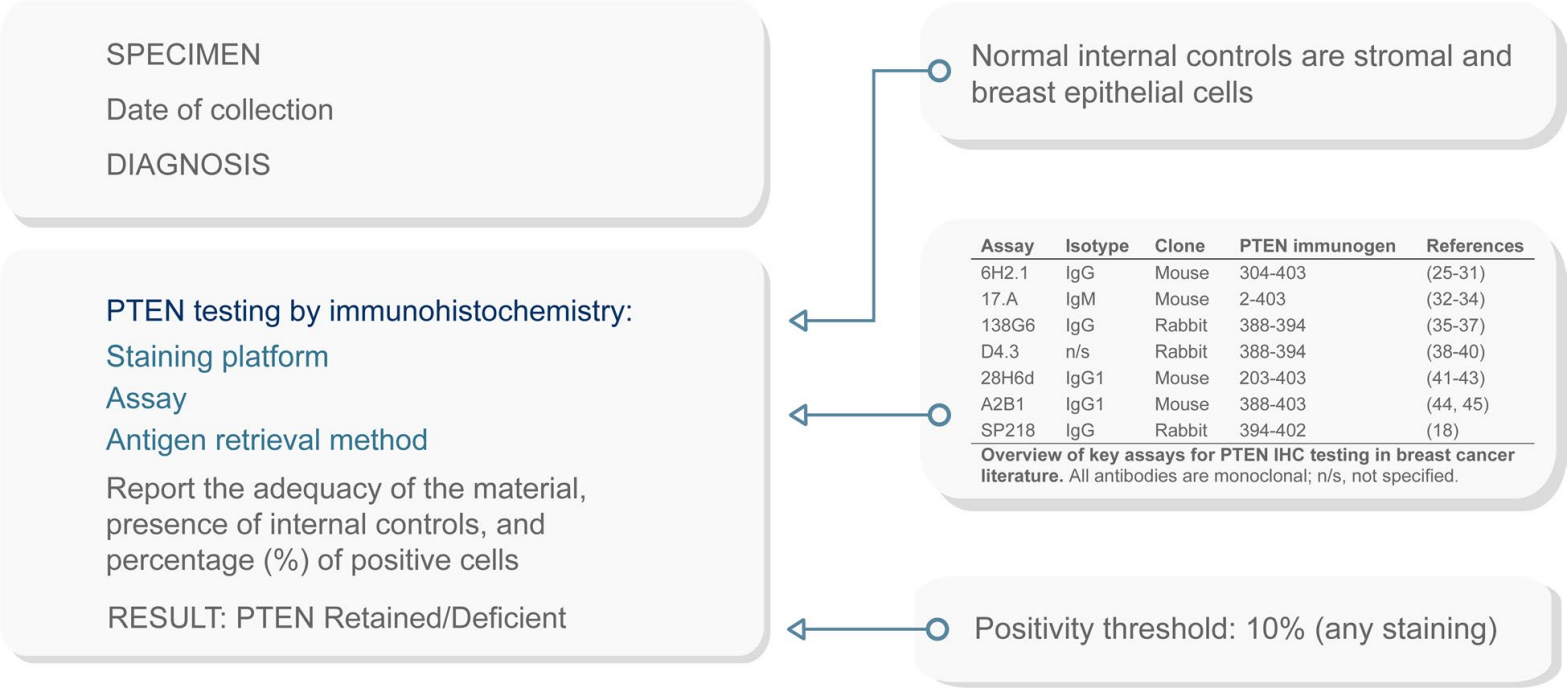
Optimized report for PTEN testing by immunohistochemistry in HR +/HER2 − metastatic breast cancer

Timely release of a clear, concise, and comprehensive report is crucial for informed clinical decision-making in breast cancer management [71, 72]. To ensure quality, strict adherence to SOPs is essential, covering the entire diagnostic workflow from specimen excision to the final IHC report [73, 74]. Although formal guidelines specific to PTEN assessment are not as established as those for other breast cancer biomarkers, such as hormone receptors, HER2, or PD-L1, laboratories are encouraged to implement rigorous quality control measures similar to those recommended for other IHC assays [75–78]. The quality of PTEN testing results depends directly on the expertise of laboratory personnel, including technicians and pathologists, who must evaluate staining intensity and the proportion of positive tumor cells using a 10% cutoff to define positivity. Methodological details, such as the primary antibody used and specifics of the staining protocol, should be clearly documented in the report along with a statement confirming adherence to established best practices. In Fig.2, we propose an optimal reporting format for PTEN testing that aims to standardize and enhance the consistency of PTEN evaluation in breast cancer.

## Conclusion

Standardized PTEN IHC testing plays a significant role in evaluating PI3K pathway alterations in HR +/HER2 − MBC, especially in laboratories without access to NGS, when archival tissue is inadequate for sequencing, or in case of pre-analytical failures, as observed in approximately 10% of samples in the CAPItello-291 trial. Thus, IHC is a feasible choice for routine clinical practice, whereas NGS is preferable.

## References

[1] Masson GR, Williams RL (2020) Structural mechanisms of PTEN regulation. Cold Spring Harb Perspect Med. 10.1101/cshperspect.a036152 31636093 10.1101/cshperspect.a036152 PMC7050585

[2] Zhan H, Antony VM, Tang H, Theriot J, Liang Y, Hui P et al (2025) *PTEN* inactivating mutations are associated with hormone receptor loss during breast cancer recurrence. Breast Cancer Res Treat. 10.1007/s10549-025-07660-3 40063317 10.1007/s10549-025-07660-3

[3] Hao C, Wei Y, Meng W, Zhang J, Yang X (2025) PI3K/AKT/mTOR inhibitors for hormone receptor-positive advanced breast cancer. Cancer Treat Rev 132:102861 39662202 10.1016/j.ctrv.2024.102861

[4] Rugo HS, Lerebours F, Ciruelos E, Drullinsky P, Ruiz-Borrego M, Neven P et al (2021) Alpelisib plus fulvestrant in PIK3CA-mutated, hormone receptor-positive advanced breast cancer after a CDK4/6 inhibitor (BYLieve): one cohort of a phase 2, multicentre, open-label, non-comparative study. Lancet Oncol 22:489–498 33794206 10.1016/S1470-2045(21)00034-6

[5] Juric D, Kalinsky K, Turner NC, Jhaveri KL, Schmid P, Loi S et al (2024) First-line inavolisib/placebo + palbociclib + fulvestrant (Inavo/Pbo+Palbo+Fulv) in patients (pts) with PIK3CA-mutated, hormone receptor-positive, HER2-negative locally advanced/metastatic breast cancer who relapsed during/within 12 months (mo) of adjuvant endocrine therapy completion: INAVO120 phase III randomized trial additional analyses. J Clin Oncol 42:1003

[6] Turner NC, Oliveira M, Howell SJ, Dalenc F, Cortes J, Gomez Moreno HL et al (2023) Capivasertib in hormone receptor-positive advanced breast cancer. N Engl J Med 388:2058–2070 37256976 10.1056/NEJMoa2214131 PMC11335038

[7] Fusco N, Malapelle U (2025) Next-generation sequencing for PTEN testing in HR+/HER2˗ metastatic breast cancer. Crit Rev Oncol Hematol 207:104626 39909182 10.1016/j.critrevonc.2025.104626

[8] Dilawari A, Buturla J, Osgood C, Gao X, Chen W, Ricks TK et al (2024) US food and drug administration approval summary: capivasertib with fulvestrant for hormone receptor-positive, human epidermal growth factor receptor 2-negative locally advanced or metastatic breast cancer with PIK3CA/AKT1/PTEN alterations. J Clin Oncol. 10.1200/JCO.24.00427 39159418 10.1200/JCO.24.00427 PMC11588547

[9] Cano Barbadilla T, Alvarez Perez M, Prieto Cuadra JD, Dawid de Vera MT, Alberca-Del Arco F, Garcia Munoz I et al (2024) The role of immunohistochemistry as a surrogate marker in molecular subtyping and classification of bladder cancer. Diagnostics (Basel) 14(22):2501 10.3390/diagnostics14222501 PMC11592502 39594166

[10] Hoda RS, Brogi E, Wen HY (2022) Quality issues in diagnostic immunohistochemistry in breast pathology. Pathobiology 89:324–333 35443240 10.1159/000522538

[11] Venetis K, Frascarelli C, Bielo LB, Cursano G, Adorisio R, Ivanova M et al (2025) Mismatch repair (MMR) and microsatellite instability (MSI) phenotypes across solid tumors: a comprehensive cBioPortal study on prevalence and prognostic impact. Eur J Cancer. 10.1016/j.ejca.2025.115233 39827722 10.1016/j.ejca.2025.115233

[12] Ivanova M, Frascarelli C, Cerbelli B, Pignataro MG, Pernazza A, Venetis K et al (2024) PD-L1 testing in metastatic triple-negative breast cancer: interobserver and interplatform reproducibility of CE-IVD assays for CPS and IC scores. Hum Pathol 144:22–27 38278450 10.1016/j.humpath.2024.01.008

[13] Fusco N, Sajjadi E, Venetis K, Gaudioso G, Lopez G, Corti C et al (2020) PTEN alterations and their role in cancer management: are we making headway on precision medicine? Genes 11:719 32605290 10.3390/genes11070719 PMC7397204

[14] Garg K, Broaddus RR, Soslow RA, Urbauer DL, Levine DA, Djordjevic B (2012) Pathological scoring of PTEN immunohistochemistry in endometrial carcinoma is highly reproducible. Int J Gynecol Pathol 31:48–56 22123723 10.1097/PGP.0b013e3182230d00 PMC4244710

[15] Rugo H, Cortes J, Oliveira M, Howell S, Dalenc F, Gomez H et al (2024) P2–03–19: capivasertib-fulvestrant for patients w/ HR-pos/HER2-negative advanced breast cancer who had relapsed or progressed during or after aromatase inhibitor treatment: exploratory analysis of PTEN deficiency by IHC from phase III CAPItello-291 trial. San Antonio Breast Cancer Symposium 2024 Abstract nr. P2–03–19

[16] Mosele MF, Westphalen CB, Stenzinger A, Barlesi F, Bayle A, Bieche I et al (2024) Recommendations for the use of next-generation sequencing (NGS) for patients with advanced cancer in 2024: a report from the ESMO precision medicine working group. Ann Oncol 35:588–606 38834388 10.1016/j.annonc.2024.04.005

[17] Venetis K, Trapani D, Adorisio R, Ranghiero A, Castellano G, Peruzzo V et al (2024) ESR1 ctDNA testing in HR+/HER2- metastatic breast cancer: a real-world perspective from a referral laboratory. The Journal of Liquid Biopsy 5:100229

[18] Liang H, Yin Y (2019) Multiple roles of PTEN isoforms PTENalpha and PTENbeta in cellular activities and tumor development. Sci China Life Sci 62:1722–1724 31823204 10.1007/s11427-019-1595-2

[19] Xu B, Alminawi S, Boulianne P, Shang YM, Downes MR, Slodkowska E (2021) The impact of pre-analytical parameters on class II biomarkers by immunohistochemistry: concordance across four tissue processing protocols. Virchows Arch 478:985–993 33175216 10.1007/s00428-020-02960-z

[20] Terra SA, de Arruda Lourencao PLT, Rodrigues MAM (2022) PTEN immunohistochemistry. Arch Pathol Lab Med 147:577–583 35943858 10.5858/arpa.2021-0424-OA

[21] Alvarez-Garcia V, Tawil Y, Wise HM, Leslie NR (2019) Mechanisms of PTEN loss in cancer: it’s all about diversity. Semin Cancer Biol 59:66–79 30738865 10.1016/j.semcancer.2019.02.001

[22] Perren A, Weng LP, Boag AH, Ziebold U, Thakore K, Dahia PL et al (1999) Immunohistochemical evidence of loss of PTEN expression in primary ductal adenocarcinomas of the breast. Am J Pathol 155:1253–1260 10514407 10.1016/S0002-9440(10)65227-3 PMC1867038

[23] Yonemori K, Tsuta K, Shimizu C, Hatanaka Y, Hashizume K, Ono M et al (2009) Immunohistochemical expression of PTEN and phosphorylated Akt are not correlated with clinical outcome in breast cancer patients treated with trastuzumab-containing neo-adjuvant chemotherapy. Med Oncol 26:344–349 19016009 10.1007/s12032-008-9127-2

[24] Dave B, Migliaccio I, Gutierrez MC, Wu MF, Chamness GC, Wong H et al (2011) Loss of phosphatase and tensin homolog or phosphoinositol-3 kinase activation and response to trastuzumab or lapatinib in human epidermal growth factor receptor 2-overexpressing locally advanced breast cancers. J Clin Oncol 29:166–173 21135276 10.1200/JCO.2009.27.7814 PMC3058274

[25] Razis E, Bobos M, Kotoula V, Eleftheraki AG, Kalofonos HP, Pavlakis K et al (2011) Evaluation of the association of *PIK3CA* mutations and *PTEN* loss with efficacy of trastuzumab therapy in metastatic breast cancer. Breast Cancer Res Treat 128:447–456 21594665 10.1007/s10549-011-1572-5

[26] Gschwantler-Kaulich D, Tan YY, Fuchs EM, Hudelist G, Kostler WJ, Reiner A et al (2017) Pten expression as a predictor for the response to trastuzumab-based therapy in Her-2 overexpressing metastatic breast cancer. PLoS ONE 12:e0172911 28253285 10.1371/journal.pone.0172911 PMC5333838

[27] Lopez G, Noale M, Corti C, Gaudioso G, Sajjadi E, Venetis K et al (2020) PTEN expression as a complementary biomarker for mismatch repair testing in breast cancer. Int J Mol Sci. 10.3390/ijms21041461. (**21**) 32098071 10.3390/ijms21041461 PMC7073136

[28] Sajjadi E, Venetis K, Piciotti R, Gambini D, Blundo C, Runza L et al (2021) Combined analysis of PTEN, HER2, and hormone receptors status: remodeling breast cancer risk profiling. BMC Cancer 21:1152 34706703 10.1186/s12885-021-08889-z PMC8555186

[29] Torres J, Navarro S, Rogla I, Ripoll F, Lluch A, Garcia-Conde J et al (2001) Heterogeneous lack of expression of the tumour suppressor PTEN protein in human neoplastic tissues. Eur J Cancer 37:114–121 11165138 10.1016/s0959-8049(00)00366-x

[30] Panigrahi AR, Pinder SE, Chan SY, Paish EC, Robertson JF, Ellis IO (2004) The role of PTEN and its signalling pathways, including AKT, in breast cancer; an assessment of relationships with other prognostic factors and with outcome. J Pathol 204:93–100 15307142 10.1002/path.1611

[31] Siddiqui S, Akhter N, Deo SV, Shukla NK, Husain SA (2016) A study on promoter methylation of PTEN in sporadic breast cancer patients from North India. Breast Cancer 23:922–931 26754093 10.1007/s12282-015-0665-0

[32] Esteva FJ, Guo H, Zhang S, Santa-Maria C, Stone S, Lanchbury JS et al (2010) PTEN, PIK3CA, p-AKT, and p-p70S6K status: association with trastuzumab response and survival in patients with HER2-positive metastatic breast cancer. Am J Pathol 177:1647–1656 20813970 10.2353/ajpath.2010.090885 PMC2947262

[33] Perez EA, Dueck AC, McCullough AE, Chen B, Geiger XJ, Jenkins RB et al (2013) Impact of PTEN protein expression on benefit from adjuvant trastuzumab in early-stage human epidermal growth factor receptor 2-positive breast cancer in the North Central Cancer Treatment Group N9831 trial. J Clin Oncol 31:2115–2122 23650412 10.1200/JCO.2012.42.2642 PMC3731983

[34] Beelen K, Opdam M, Severson TM, Koornstra RH, Vincent AD, Wesseling J et al (2014) PIK3CA mutations, phosphatase and tensin homolog, human epidermal growth factor receptor 2, and insulin-like growth factor 1 receptor and adjuvant tamoxifen resistance in postmenopausal breast cancer patients. Breast Cancer Res 16:R13 24467828 10.1186/bcr3606 PMC3978618

[35] Loibl S, Darb-Esfahani S, Huober J, Klimowicz A, Furlanetto J, Lederer B et al (2016) Integrated analysis of PTEN and p4EBP1 protein expression as predictors for pCR in HER2-positive breast cancer. Clin Cancer Res 22:2675–2683 26758558 10.1158/1078-0432.CCR-15-0965

[36] Wang LL, Hao S, Zhang S, Guo LJ, Hu CY, Zhang G et al (2017) PTEN/PI3K/AKT protein expression is related to clinicopathological features and prognosis in breast cancer with axillary lymph node metastases. Hum Pathol 61:49–57 27864123 10.1016/j.humpath.2016.07.040

[37] Rimawi MF, De Angelis C, Contreras A, Pareja F, Geyer FC, Burke KA et al (2018) Low PTEN levels and PIK3CA mutations predict resistance to neoadjuvant lapatinib and trastuzumab without chemotherapy in patients with HER2 over-expressing breast cancer. Breast Cancer Res Treat 167:731–740 29110152 10.1007/s10549-017-4533-9 PMC5821069

[38] Bakarakos P, Theohari I, Nomikos A, Mylona E, Papadimitriou C, Dimopoulos AM et al (2010) Immunohistochemical study of PTEN and phosphorylated mTOR proteins in familial and sporadic invasive breast carcinomas. Histopathology 56:876–882 20636791 10.1111/j.1365-2559.2010.03570.x

[39] Fabi A, Metro G, Di Benedetto A, Nistico C, Vici P, Melucci E et al (2010) Clinical significance of PTEN and p-Akt co-expression in HER2-positive metastatic breast cancer patients treated with trastuzumab-based therapies. Oncology 78:141–149 20389136 10.1159/000312656

[40] Duman BB, Sahin B, Acikalin A, Ergin M, Zorludemir S (2013) PTEN, Akt, MAPK, p53 and p95 expression to predict trastuzumab resistance in HER2 positive breast cancer. J BUON 18:44–50 23613387

[41] Depowski PL, Rosenthal SI, Ross JS (2001) Loss of expression of the PTEN gene protein product is associated with poor outcome in breast cancer. Mod Pathol 14:672–676 11454999 10.1038/modpathol.3880371

[42] Capodanno A, Camerini A, Orlandini C, Baldini E, Resta ML, Bevilacqua G et al (2009) Dysregulated PI3K/Akt/PTEN pathway is a marker of a short disease-free survival in node-negative breast carcinoma. Hum Pathol 40:1408–1417 19428048 10.1016/j.humpath.2009.02.005

[43] Tokunaga E, Iwata H, Itoh M, Taira T, Toyama T, Mizuno T et al (2025) Capivasertib and fulvestrant for patients with HR-positive/HER2-negative advanced breast cancer: analysis of the subgroup of patients from Japan in the phase 3 CAPItello-291 trial. Breast Cancer 32:132–143 39379782 10.1007/s12282-024-01640-z PMC11717841

[44] Bussolati G, Annaratone L, Maletta F (2015) The pre-analytical phase in surgical pathology. Recent Results Cancer Res 199:1–13 25636424 10.1007/978-3-319-13957-9_1

[45] Badve SS, Gokmen-Polar Y (2022) Protein profiling of breast cancer for treatment decision-making. Am Soc Clin Oncol Educ Book 42:1–9 35580295 10.1200/EDBK_351207

[46] Fusco N, Ragazzi M, Sajjadi E, Venetis K, Piciotti R, Morganti S et al (2021) Assessment of estrogen receptor low positive status in breast cancer: implications for pathologists and oncologists. Histol Histopathol 36(12):18376 10.14670/HH-18-376 34585734

[47] Sajjadi E, Venetis K, Ivanova M, Fusco N (2022) Improving HER2 testing reproducibility in HER2-low breast cancer. Cancer Drug Resist 5:882–888 36627898 10.20517/cdr.2022.29 PMC9771736

[48] Ghlichloo I, Shi WJ, Fadare O (2025) The effect of prolonged cold ischemia time on breast cancer biomarker expression after neoadjuvant chemotherapy. Pathology 266:155781 10.1016/j.prp.2024.155781 39709874

[49] Ma X, Zhou L, Wu Q, Jia L, Diao X, Kang Q et al (2023) Loss of human epidermal receptor 2 expression in formalin-fixed paraffin-embedded breast cancer samples and the rescuing effect of enhanced antigen retrieval and signal amplification. Life (Basel). 10.3390/life14010031 38255647 10.3390/life14010031 PMC10820269

[50] Stumptner C, Pabst D, Loibner M, Viertler C, Zatloukal K (2019) The impact of crosslinking and non-crosslinking fixatives on antigen retrieval and immunohistochemistry. N Biotechnol 52:69–83 31082574 10.1016/j.nbt.2019.05.003

[51] Scalia CR, Boi G, Bolognesi MM, Riva L, Manzoni M, DeSmedt L et al (2017) Antigen masking during fixation and embedding, dissected. J Histochem Cytochem 65:5–20 27798289 10.1369/0022155416673995 PMC5256198

[52] Wang J, Yoon E, Krishnamurthy S (2024) Concordance between pathologists and between specimen types in detection of HER2-low breast carcinoma by immunohistochemistry. Ann Diagn Pathol 70:152288 38452457 10.1016/j.anndiagpath.2024.152288

[53] Guerini Rocco E, Eccher A, Girolami I, Graziano P, Fontanini G, Vigliar E et al (2022) Concordance between three PD-L1 immunohistochemical assays in head and neck squamous cell carcinoma (HNSCC) in a multicenter study. Diagnostics (Basel) 12:12 10.3390/diagnostics12020477 PMC8871075 35204568

[54] Noske A, Wagner D-C, Schwamborn K, Foersch S, Steiger K, Kiechle M et al (2021) Interassay and interobserver comparability study of four programmed death-ligand 1 (PD-L1) immunohistochemistry assays in triple-negative breast cancer. Breast 60:238–244 34768219 10.1016/j.breast.2021.11.003 PMC8602040

[55] Bazzichetto C, Conciatori F, Pallocca M, Falcone I, Fanciulli M, Cognetti F et al (2019) PTEN as a prognostic/predictive biomarker in cancer: an unfulfilled promise? Cancers (Basel). 10.3390/cancers11040435 30925702 10.3390/cancers11040435 PMC6520939

[56] Dameri M, Cirmena G, Ravera F, Ferrando L, Cuccarolo P, Stabile M et al (2023) Standard operating procedures (SOPs) for non-invasive multiple biomarkers detection in an academic setting: a critical review of the literature for the RENOVATE study protocol. Critical Reviews in Oncology/Hematology 185:103963 36931614 10.1016/j.critrevonc.2023.103963

[57] Bonizzi G, Capra M, Cassi C, Taliento G, Pala O, Sajjadi E et al (2022) Biobank for translational medicine: standard operating procedures for optimal sample management. J Vis Exp 2022(189):1-5 10.3791/63950 36533819

[58] Fusco N, Malapelle U, Rolfo C (2025) Role of the International Society of Liquid Biopsy (ISLB) in establishing quality control frameworks for clinical integration. Crit Rev Oncol Hematol. 10.1016/j.critrevonc.2025.104619 39824370 10.1016/j.critrevonc.2025.104619

[59] Esposito A, Crimini E, Criscitiello C, Belli C, Scafetta R, Scalia R et al (2024) The safety and suitability of DNA sequencing of tissue biopsies performed on patients referred to a phase I unit. Cancers (Basel) 16:4252 10.3390/cancers16244252 PMC11674575 39766151

[60] Guerini-Rocco E, Venetis K, Cursano G, Mane E, Frascarelli C, Pepe F et al (2024) Standardized molecular pathology workflow for ctDNA-based ESR1 testing in HR+/HER2- metastatic breast cancer. Crit Rev Oncol Hematol 201:104427 38917944 10.1016/j.critrevonc.2024.104427

[61] Venetis K, Pepe F, Pescia C, Cursano G, Criscitiello C, Frascarelli C et al (2023) ESR1 mutations in HR+/HER2-metastatic breast cancer: enhancing the accuracy of ctDNA testing. Cancer Treat Rev 121:102642 37864956 10.1016/j.ctrv.2023.102642

[62] Corradi A, Bonizzi G, Sajjadi E, Pavan F, Fumagalli M, Molendini LO et al (2025) The regulatory landscape of biobanks in Europe: from accreditation to intellectual property. Curr Genomics 26:15–23 39931203 10.2174/0113892029313697240729091922 PMC11808582

[63] Nielsen S, Bzorek M, Vyberg M, Roge R (2023) Lessons learned, challenges taken, and actions made for “precision” immunohistochemistry. Analysis and Perspectives From the NordiQC Proficiency Testing Program. Appl Immunohistochem Mol Morphol 31:452–458 36194495 10.1097/PAI.0000000000001071 PMC10396077

[64] Vyberg M, Nielsen S (2016) Proficiency testing in immunohistochemistry–experiences from Nordic Immunohistochemical Quality Control (NordiQC). Virchows Arch 468:19–29 26306713 10.1007/s00428-015-1829-1 PMC4751198

[65] Buchta C, Marrington R, De la Salle B, Albarede S, Badrick T, Bietenbeck A et al (2025) Behind the scenes of EQA - characteristics, capabilities, benefits and assets of external quality assessment (EQA). Clin Chem Lab Med 63(5):879–897 10.1515/cclm-2024-1292 39754494

[66] Dodson A, Parry S, Lissenberg-Witte B, Haragan A, Allen D, O’Grady A et al (2020) External quality assessment demonstrates that PD-L1 22C3 and SP263 assays are systematically different. J Pathol Clin Res 6:138–145 31849189 10.1002/cjp2.153 PMC7164369

[67] Fusco N, Venetis K, Pepe F, Shetty O, Farinas SC, Heeke S et al (2025) International society of liquid biopsy (ISLB) perspective on minimal requirements for ctDNA testing in solid tumors. The Journal of Liquid Biopsy 8:100301 40521567 10.1016/j.jlb.2025.100301 PMC12164225

[68] Pulido R, Mingo J, Gaafar A, Nunes-Xavier CE, Luna S, Torices L et al (2019) Precise immunodetection of PTEN protein in human neoplasia. Cold Spring Harb Perspect Med. 10.1101/cshperspect.a036293 31501265 10.1101/cshperspect.a036293 PMC6886455

[69] Sakr RA, Barbashina V, Morrogh M, Chandarlapaty S, Andrade VP, Arroyo CD et al (2010) Protocol for PTEN expression by immunohistochemistry in formalin-fixed paraffin-embedded human breast carcinoma. Appl Immunohistochem Mol Morphol 18:371–374 20216404 10.1097/PAI.0b013e3181d50bd5 PMC2921801

[70] Rugo HS, Oliveira M, Howell SJ, Dalenc F, Cortes J, Gomez HL et al (2024) Capivasertib and fulvestrant for patients with hormone receptor-positive advanced breast cancer: characterization, time course, and management of frequent adverse events from the phase III CAPItello-291 study. ESMO Open 9:103697 39241495 10.1016/j.esmoop.2024.103697 PMC11406080

[71] Boscolo Bielo L, Guerini Rocco E, Crimini E, Repetto M, Lombardi M, Zanzottera C et al (2025) Molecular tumor board in patients with metastatic breast cancer. Breast Cancer Res Treat 210:45–55 39476312 10.1007/s10549-024-07535-z

[72] Pepe F, Venetis K, Cursano G, Frascarelli C, Pisapia P, Vacirca D et al (2024) PIK3CA testing in hormone receptor-positive/HER2-negative metastatic breast cancer: real-world data from Italian molecular pathology laboratories. Pharmacogenomics 25:161–169 38440825 10.2217/pgs-2023-0238

[73] Ivanova M, Porta FM, D'Ercole M, Pescia C, Sajjadi E, Cursano G et al (2023) Standardized pathology report for HER2 testing in compliance with 2023 ASCO/CAP updates and 2023 ESMO consensus statements on HER2-low breast cancer. Virchows Arch 484(1):3–14 10.1007/s00428-023-03656-w PMC10791807 37770765

[74] Tan PH, Mihir G, Laokulrath N, Rakha E (2024) Practical approach to scoring HER2 immunohistochemistry in breast cancer in the wake of updated guidelines. Histopathology 84:715–718 38087653 10.1111/his.15117

[75] Fusco N, Viale G (2024) The, “lows”: update on ER-low and HER2-low breast cancer. Breast 78:103831 39486153 10.1016/j.breast.2024.103831 PMC11564046

[76] Fusco N, Ivanova M, Frascarelli C, Criscitiello C, Cerbelli B, Pignataro MG et al (2023) Advancing the PD-L1 CPS test in metastatic TNBC: insights from pathologists and findings from a nationwide survey. Crit Rev Oncol Hematol 190:104103 37595344 10.1016/j.critrevonc.2023.104103

[77] Bogen SA, Dabbs DJ, Miller KD, Nielsen S, Parry SC, Szabolcs MJ et al (2022) A consortium for analytic standardization in immunohistochemistry. Arch Pathol Lab Med 147:584–590 36084252 10.5858/arpa.2022-0031-RA PMC11681772

[78] Katcher AH, Greenman MP, Roychoudhury S, Goldberg GL (2024) Utilization of immunohistochemistry in gynecologic tumors: an expert review. Gynecol Oncol Rep 56:101550 39717157 10.1016/j.gore.2024.101550 PMC11664289

